# Statistical Forecast of Pollution Episodes in Macao during National Holiday and COVID-19

**DOI:** 10.3390/ijerph17145124

**Published:** 2020-07-15

**Authors:** Man Tat Lei, Joana Monjardino, Luisa Mendes, David Gonçalves, Francisco Ferreira

**Affiliations:** 1 Department of Sciences and Environmental Engineering, NOVA School of Science and Technology, NOVA University Lisbon, 2829-516 Caparica, Portugal; lc.mendes@fct.unl.pt; 2 Institute of Science and Environment, University of Saint Joseph, Macau 999078, China; david.goncalves@usj.edu.mo; 3 Center for Environmental and Sustainability Research, NOVA School of Science and Technology, NOVA University Lisbon, 2829-516 Caparica, Portugal; jvm@fct.unl.pt (J.M.); ff@fct.unl.pt (F.F.)

**Keywords:** air pollution, air quality forecast, modelling, pollution episodes, national holiday, COVID-19

## Abstract

Statistical methods such as multiple linear regression (MLR) and classification and regression tree (CART) analysis were used to build prediction models for the levels of pollutant concentrations in Macao using meteorological and air quality historical data to three periods: (i) from 2013 to 2016, (ii) from 2015 to 2018, and (iii) from 2013 to 2018. The variables retained by the models were identical for nitrogen dioxide (NO_2_), particulate matter (PM_10_), PM_2.5_, but not for ozone (O_3_) Air pollution data from 2019 was used for validation purposes. The model for the 2013 to 2018 period was the one that performed best in prediction of the next-day concentrations levels in 2019, with high coefficient of determination (R^2^), between predicted and observed daily average concentrations (between 0.78 and 0.89 for all pollutants), and low root mean square error (RMSE), mean absolute error (MAE), and biases (BIAS). To understand if the prediction model was robust to extreme variations in pollutants concentration, a test was performed under the circumstances of a high pollution episode for PM_2.5_ and O_3_ during 2019, and the low pollution episode during the period of implementation of the preventive measures for COVID-19 pandemic. Regarding the high pollution episode, the period of the Chinese National Holiday of 2019 was selected, in which high concentration levels were identified for PM_2.5_ and O_3_, with peaks of daily concentration exceeding 55 μg/m^3^ and 400 μg/m^3^, respectively. The 2013 to 2018 model successfully predicted this high pollution episode with high coefficients of determination (of 0.92 for PM_2.5_ and 0.82 for O_3_). The low pollution episode for PM_2.5_ and O_3_ was identified during the 2020 COVID-19 pandemic period, with a low record of daily concentration for PM_2.5_ levels at 2 μg/m^3^ and O_3_ levels at 50 μg/m^3^, respectively. The 2013 to 2018 model successfully predicted the low pollution episode for PM_2.5_ and O_3_ with a high coefficient of determination (0.86 and 0.84, respectively). Overall, the results demonstrate that the statistical forecast model is robust and able to correctly reproduce extreme air pollution events of both high and low concentration levels.

## 1. Introduction

The development of air quality forecast models is essential for cities with high population density, including Macao, one of the most densely populated cities in the world. It is extremely important to predict pollution episodes so the authority can provide a warning to the local community in advance to avoid the adverse air quality, which may lead to severe health consequences. In order to predict next-day concentrations of nitrogen dioxide (NO_2_), particulate matter (PM_10_ and PM_2.5_), and maximum hourly concentration of ozone (O_3 MAX_) for roadside, ambient, and residential stations in Macao, a forecast model was developed based on statistical methods using multiple linear regression (MLR) and classification and regression tree (CART) analysis.

There are three forms of total suspended particles (TSPs), which include coarse, fine, and ultrafine particles. Coarse particles, also known as PM_10_, are derived from suspension of dust, soil, sea salts, pollen, mold, and other crustal materials. Fine particles, also known as PM_2.5_, are derived from emissions from combustion process, including vehicles powered by petrol and diesel, wood burning, coal burning, and other industrial processes. Ultrafine particles are derived from combustion related sources such as vehicle exhausts and atmospheric photochemical reactions [1].

O_3_ is the most important index substance for photochemical smog, one of the major air pollutants [2]. The formation of ground-level O_3_ heavily depends on the concentration levels of volatile organic compounds (VOCs) and nitrogen oxides (NO_x_) and meteorological factors such as wind speed, insolation, and temperature. PM_2.5_ and O_3_ pollutants are known to cause the most damages to the human respiratory and cardiovascular system. A study for Terengganu State, Malaysia, showed that high levels of O_3_ occurring under dry and warm conditions during the southwest monsoon, were higher in industrial areas, and were positively correlated with the maximum daily temperature [3].

The emission of NO_x_ is primarily emitted from transportation and combustion process, while the emission of VOCs is primarily emitted from road traffic and the use of products containing organic solvents [4,5].

The emission of NO_x_ and VOCs is responsible for the O_3_ formation, in particular rural areas being NO_x_-sensitive while urban areas being VOC-sensitive. Nevertheless, the greater NO_x_ emission reductions have contributed to a widespread shift in the O_3_ production regime from NO_x_-saturated (high-NO_x_) to NO_x_-sensitive (low-NO_x_) in some urban areas, while O_3_ production in rural areas is even more sensitive to NO_x_.

TSPs are primary contributors to premature death worldwide, with over four million premature deaths being recorded due to exposure to high levels of ambient PM_2.5_ [6,7,8]. PM_2.5_ can penetrate deep into the lungs when being inhaled, which leads to both acute and chronic health issues [1,6]. NO_2_ and TSPs are responsible for 412,000 and 71,000 premature death per year, respectively, in the European Union [9,10]. Moreover, previous studies show a strong correlation between short-term exposure to NO_2_ and both the number of hospital outpatients with eye and adnexa diseases (EADs) [11] and the number of hospital admission due to cardiovascular diseases (CVD) [12]. The Chinese National Ambient Air Quality Standard (NAAQS) has set the threshold of PM_10_, PM_2.5_, and O_3 MAX_ concentration at 150 μg/m^3^, 75 μg/m^3^, and 160 μg/m^3^, respectively, while the WHO Air Quality Guideline has set the same thresholds at 50 μg/m^3^, 25 μg/m^3^, and 100 μg/m^3^, respectively. Compliance with the thresholds set by the WHO for PM_2.5_ could improve life expectancy in China by 0.14 years [13] and ambient air pollution has caused at least 3.7 million deaths, with more than 25% of deaths in Southeast Asia [14,15].

Air pollution forecasting models can provide important information for populations to adopt mitigation measures during high pollution days. To be useful, these models should be robust to deal with extreme variations in pollution levels, in particular during high-pollution peak days. Factors leading to extreme variation in pollution levels are diverse and include both human activities and meteorological factors.

In a study for Beijing, China, the reduction of traffic flow and vehicle emissions in downtown areas during the Chinese National Holiday, reduced air pollution, while, in contrast, fireworks during the Chinese New Year Holiday had the opposite effect [16]. When highway tolls were being waived for passenger vehicles during the Chinese National Holiday across the entire nation of China, air pollution increased by 20% and visibility decreased by 1 km, causing economic losses due to negative health impacts estimated at RMB 0.95 billion [17]. Nevertheless, the Chinese National Holiday is known to be a golden week of tourism, in which the Chinese tourist flock to different tourist destinations around the world to celebrate the national holiday. Due to the vibrant casinos and entertainment industry and close proximity to mainland China, Macao is also one of the favorite destinations for Chinese tourists, so the influx of tourist during the period of Chinese National Holiday may lead to an increase of emissions in Macao.

Likewise, the recent COVID-19 crisis has had an extreme impact in air pollution levels. The Wuhan Health Commission has first reported cases of pneumonia linked to the Wuhan wet market in Hubei Province, China, back in December 2019 [18]. Preventive measures were implemented soon after that abruptly reduced industrial activities and transportation. Nevertheless, the levels of air pollutants, in particular of PM_2.5_, remained severe in northern China throughout the end of January 2020 due to adverse meteorological conditions that have overwhelmed the benefits of emission reduction in transportation and industrial sectors [19].

Previous work showed that there is an increase in the level of O_3_ concentrations and a decrease in the level of NO_2_, PM_10_, and PM_2.5_ concentration during the period of COVID-19 pandemic lockdown in several cities of China, due to the significant reduction of transportation and industrial activities [4,5,20,21].

Several methodologies have been developed and applied to forecast air quality across the world, including deterministic, statistical, and machine learning methods [22,23,24,25,26]. Some studies showed that statistical models are more accurate and efficient compared to deterministic models, particularly in regions with complexed terrain [27,28,29,30] Moreover, prediction of NO_2_, PM_10_, PM_2.5_, and O_3 MAX_ concentrations based on MLR and CART models have been successfully implemented in Macao, Bangkok, Changsha City, Beijing, Bilbao, and Pakistan [26,31,32,33,34,35].

In this context, it is relevant to develop a reliable methodology to forecast the concentration of air pollutants, which is presented and tested for a high pollution episode (associated with the Chinese National Holiday) and a low pollution episode (during COVID-19 preventive measures).

## 2. Materials and Methods

The air quality and meteorological variables that were considered to build all of the air quality statistical models were obtained from Macao Meteorological and Geophysical Bureau (SMG). The air quality data was gathered from the air quality monitoring network, namely for: Macao Roadside, Macao Residential, Taipa Ambient, Taipa Residential, and Coloane Ambient stations, which have a suitable historic dataset of surface air quality measurements for the levels of NO_2_, PM_10_, PM_2.5_, and O_3_ concentrations. These background stations (residential and ambient) can capture the regional contribution of PM_10_ and PM_2.5_. There is a higher population and traffic density in Macao Roadside and Macao Residential, which are located in the main peninsula, in comparison to Taipa Ambient, Taipa Residential, and Coloane Ambient stations, which are located on the outlying islands.

Meteorological data was obtained from surface observations at SMG’s Taipa Grande Meteorological Station, hourly observations from automatic weather stations, such as temperature, relative humidity, precipitation, average wind speed, and dew point temperature, as well as upper-air observations (from Hong Kong King’s Park location) such as geopotential heights, thickness, stability, temperature, relative humidity, and dew point temperature at various altitudes. In the present work, statistical models such as multiple linear regression (MLR), and classification and regression tree (CART), are developed, based on historical measurements of meteorological and air quality variables. Table 1 presents all the variables considered as predictors in the MLR and CART forecast models, as shown in previous work [22]. The air quality variables considered included the levels of NO_2_, PM_10_, PM_2.5_, and O_3 MAX_ concentration from 00:00 to 23:00 of the previous day, two days and three days ago, and from 16:00 of the previous day and 15:00 of today. The meteorological variables being considered included the upper-air observations from King’s Park location, Hong Kong Observatory, surface observations and other variables from the monitoring network of Macao Meteorological and Geophysical Bureau (SMG).

In this study, meteorological and air quality variables for 2013 to 2016, 2015 to 2018, and 2013 to 2018 were used to build three separate forecasting models. The 2013 to 2016 model was constructed for the initial evaluation for the application of the statistical model to forecast air quality in Macao, while the 2015 to 2018 models and the 2013 to 2018 models are a follow-up, to determine if any improvement could be made with two additional years of data. The comparison of extended data ranging from 5 to 6 years are considered to be adequate lengths to test if there is any significant difference between the time series. Simultaneously, it would not be ideal to trace back too far with the time series, because regional emissions are constantly changing, and therefore the level of pollutants concentration may also be changing. The dataset from 2019 was the most recent dataset, which would be used for the model validation for all the models. This study is an empirical approach and also region-specific, which may also be chemical-regime dependent.

The final selected variables to predict the levels of PM_2.5_ and O_3_ concentration are common to different locations of Macao air quality monitoring stations. Some variables initially selected were rejected from the forecast models due to collinearity. The final objective is to obtain prediction models with the lowest number of variables, but with the maximum explained variance as translated by the coefficient of determination (R^2^).

After selecting the best model, it was applied to forecast pollution levels during an extremely high pollution episode, and a low pollution period. The high and low pollution selected episodes were, respectively: (i) the period of Chinese National Holiday, a week before the Chinese National Holiday from September 23rd to 30th, 2019, and the week during the Chinese National Holiday from October 1st to 7th, and (ii) the preventive measures period of COVID-19, from February 5th to 20th, 2020.

The statistical model was built using IBM SPSS Statistics version 26 with MLR (stepwise) and CART methods [26,36]. SPSS is a statistical software that is applied to solve research problems through hypothesis testing and predictive analysis.

Model performance indicators were calculated, such as, coefficient of determination (R^2^), root mean square error (RMSE), mean absolute error (MAE), and systematic error (BIAS).

## 3. Results and Discussion

### 3.1. Air Quality Forecast Models

The model performance indicators obtained for the 2013 to 2016 model and for the 2013 to 2018 model, validated with 2019 data, are listed in Table 2 and Table 3, respectively. The models chosen to figure in Table 2 and Table 3 are the ones that performed the worst and best 2019 validation results.

The results showed that the model for the 2013 to 2018 period was the one that performed best in predicting next-day concentrations levels in 2019, with high R^2^ between predicted and observed daily average concentrations (between 0.78 and 0.89 for all pollutants) and low RMSE, MAE, and BIAS. The additional two years of data helped to improve the air quality forecasting model. Nevertheless, with the two other models (2013–2016 and 2015–2018) a significant R^2^ (between 0.78 and 0.89 for all pollutants) was also obtained, but it translated into a less reliable air quality forecast.

Regarding model performance indicators obtained per pollutant and station, the majority of models show a good agreement and a similar R^2^ range values (from 0.81 to 0.89), except for O_3 MAX_, which is more difficult to predict. MLR was used for all pollutants, while CART analysis was used in almost all the O_3 MAX_ models (Table 2 and Table 3). This CART analysis complement was an approach to obtain improved results, mainly regarding a better prediction of high pollutant levels.

Table 4 presents the final model equations obtained for each pollutant, per air quality monitoring station, in the 2013 to 2018 model. Additionally, the final equations used to predict the levels of NO_2_, PM_10_, PM_2.5_, and O_3_MAX_ concentrations are presented in Table 4.

### 3.2. Air Quality During the High Pollution Episode

Taipa Ambient is the representative background location for Macao, and was chosen to assess the background levels of PM_2.5_ and O_3_ during the extreme pollution episode.

The influx of tourists coming to Macao, in light of the Chinese National Holiday, contributed to an high pollution episode that occurred during late September and early October 2019, with peak daily levels of PM_2.5_ concentration exceeding 55 μg/m^3^ and O_3 MAX_ levels exceeding 400 μg/m^3^, largely exceeding the threshold level recommended by the WHO.

**Figure 1:**
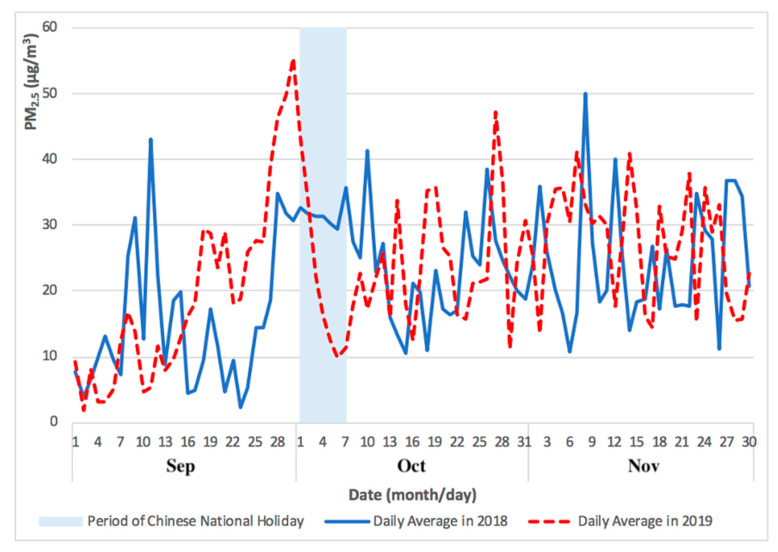
PM_2.5_ concentrations for Taipa Ambient highlighting a pollution episode immediately before, and during, the Chinese National Holiday of 2018 and 2019 (September to November).

**Figure 2:**
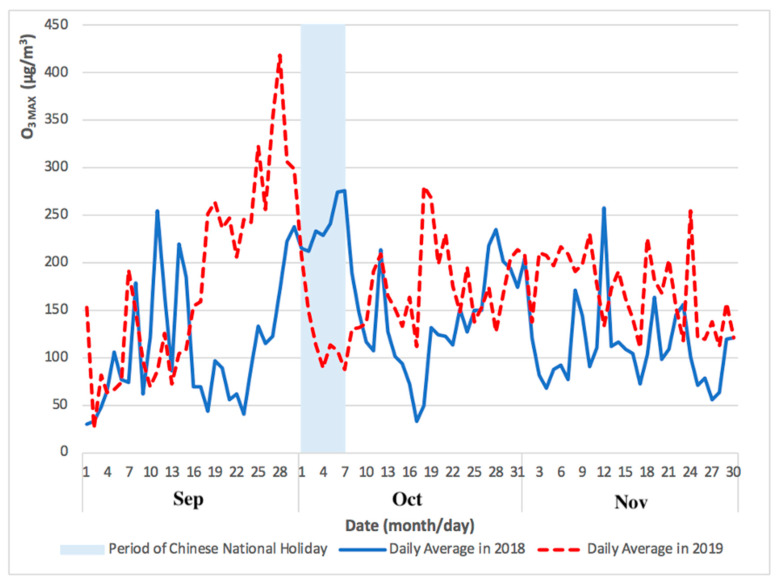
O_3 MAX_ concentrations for Taipa Ambient highlighting a pollution episode immediately before, and during, the Chinese National Holiday of 2018 and 2019 (September to November).

The levels of PM_2.5_ and O_3 MAX_ concentrations for Taipa Ambient during the Chinese National Holiday in 2019 (from September to November) are presented in Figure 1 and Figure 2.

Figure 1 and Figure 2 showed the comparison of daily average PM_2.5_ and O_3 MAX_ concentration during 2018 and 2019, from a month before in September and a month after in November of the Chinese National Holiday. The pollution episode of 2019 occurred just before and going well into the period of Chinese National Holiday (1 to 7 October).

As shown in Figure 1 and Figure 2, the levels of PM_2.5_ and O_3 MAX_ concentration peaked immediately before, and during, the Chinese National Holiday in late September and early October 2019. The monthly mean concentration of PM_2.5_ (from September to November) during the Chinese National Holiday in 2019 was 19 μg/m^3^, 24 μg/m^3^, and 28 μg/m^3^, respectively. In addition, the monthly mean concentration of O_3 MAX_ (from September to November) during the Chinese National Holiday in 2019 was 181 μg/m^3^, 163 μg/m^3^, and 172 μg/m^3^, respectively.

The levels of O_3 MAX_ concentrations reached its peak during the late September and early October due to meteorological factors including predominant winds from the north and east, from the Guangdong Province and Hong Kong, respectively. Temperatures were high in conjunction with low wind speed. The average daily temperature during the ozone peak episode that took place the two-weeks before the Chinese National Holiday (October 1st) was 28 °C, while the maximum daily average was 31 °C. Average wind speed was 2.5 m/s.

Due to the shutdown of nearby industrial sectors during the period of Chinese National Holiday, there were lower emissions of nitrogen oxides associated with the decreased load from the coal power plants in the northern region, usually supporting the operation of the factories. Therefore, this caused a decrease NO_x_, the precursor of O_3._ However, the increase in emissions of VOCs and NOx by vehicles, with chemical reactions in the presence of sunlight, may have caused the peak levels of ozone concentrations under these high temperature favorable conditions.

### 3.3. Air Quality During the Low Pollution Episode

In contrast, the COVID-19 pandemic has led to the Macao government’s decision to temporarily suspend the operation of the casinos and entertainment industry and highly restrict cross border movements, as a preventive measure to reduce population mobility within the region of Macao. As a result, it has caused a low pollution episode during late January and early February 2020, with daily levels of PM_2.5_ concentration reaching a record low at 2 μg/m^3^ and O_3 MAX_ levels at 50 μg/m^3^. The reduction of population mobility, and consequently, of traffic emissions in Macao and its nearby Guangdong Province, lead to this lowest PM_2.5_ concentration levels.

**Figure 3:**
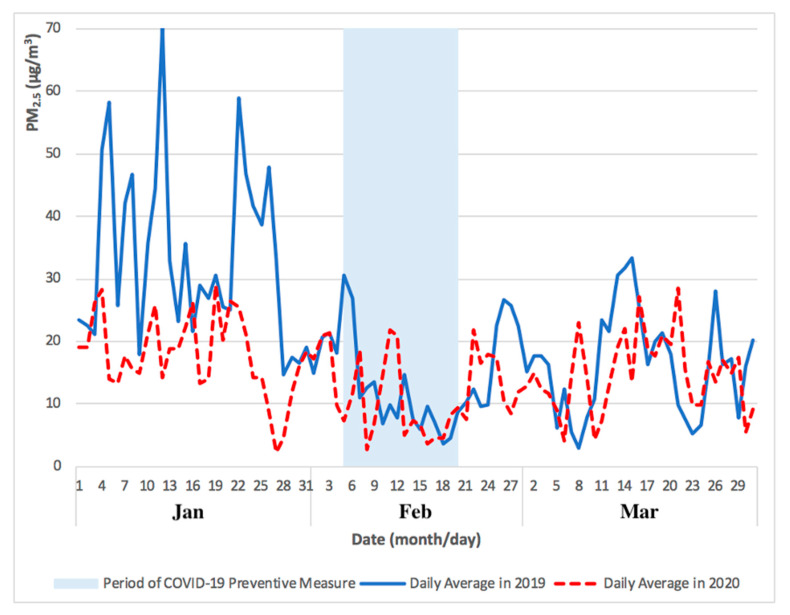
Comparison of PM_2.5_ concentrations for Taipa Ambient during the previous year of 2019 and COVID-19 pandemic in 2020 (January to March).

**Figure 4:**
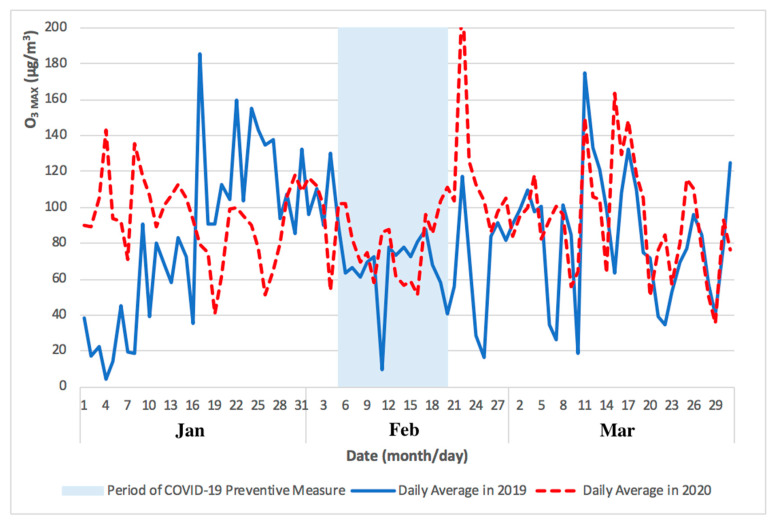
Comparison of O_3 MAX_ concentrations for Taipa Ambient during the previous year of 2019 and COVID-19 pandemic in 2020 (January to March).

As shown in Figure 3, the levels of PM_2.5_ concentrations remained low during the initial outbreak of COVID-19 pandemic in Macao (from January to February 2020), slowly recovering to pre-COVID-19 values in March 2020. As shown in Figure 4, the levels of O_3 MAX_ concentration remained high during the initial outbreak of COVID-19 pandemic in Macao (from January to February 2020) and the high levels continued into March 2020. The higher levels of O_3 MAX_ concentration were associated with lower NO_X_ emissions, which led to a weakened O_3_ titration by NO during the COVID-19 pandemic lockdown in the nearby Guangdong Province [4].

Despite industrial emission being a major contributor to the PM_2.5_ pollution in China prior to COVID-19 pandemic lockdown period, the residential emission contributed to 39% of total PM_2.5_ emissions in China, so the emissions of PM_2.5_ during the lockdown period may have originated from residential areas [5].

The comparison of PM_2.5_ and O_3 MAX_ concentrations for Taipa Ambient during the previous year of 2019 and COVID-19 pandemic in 2020 (January to March) is presented in Figure 3 and Figure 4.

**Figure 5:**
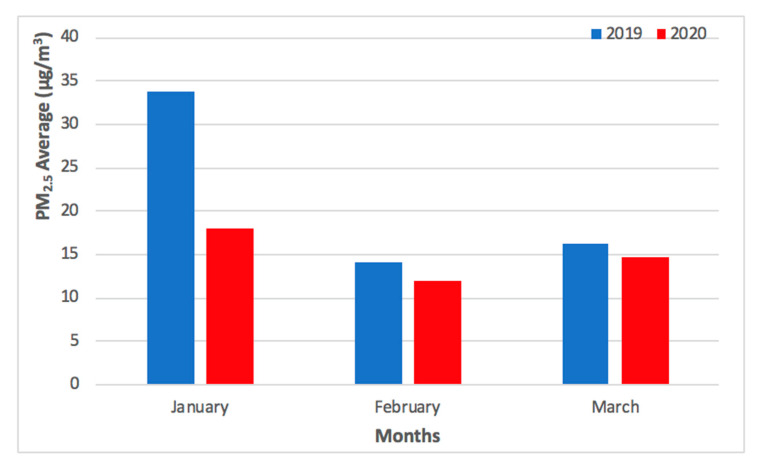
Monthly mean PM_2.5_ concentrations for Taipa Ambient during the previous year of 2019 and COVID-19 pandemic in 2020 (January to March).

**Figure 6:**
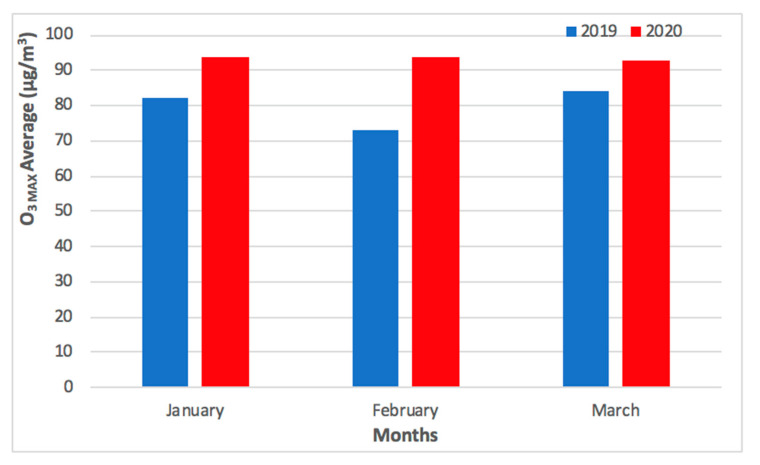
Monthly mean O_3 MAX_ concentrations for Taipa Ambient during the previous year of 2019 and COVID-19 pandemic in 2020 (January to March).

As shown in Figure 5, the difference between monthly mean concentration (from January to March) of PM_2.5_ concentration in 2019 and 2020 was 16 μg/m^3^, 2 μg/m^3^, and 1 μg/m^3^, respectively. As shown in Figure 6, the difference between monthly mean concentration (from January to March) of O_3 MAX_ concentration in 2019 and 2020 was 12 μg/m^3^, 21 μg/m^3^, and 9 μg/m^3^, respectively.

The monthly mean concentration of PM_2.5_ and O_3 MAX_ concentration for Taipa Ambient during the previous year of 2019 and COVID-19 pandemic in 2020 (January to March) is presented in Figure 5 and Figure 6. Overall, the preventive measures of COVID-19 pandemic may not have caused a significant difference in the levels of PM_2.5_ and O_3_ concentration in Macao, as the levels from February to March 2020 were similar to that of the previous year, 2019.

### 3.4. Air Quality Pollution Episodes Discussion

The air quality of Macao, a territory with only 32.8 km^2^, is heavily influenced by external factors, in particular by human activities that occur in the much larger and neighboring Guangdong province. Our study shows the extent to which an increase in mobility associated with Chinese National Holiday, or a decrease in the same factors, associated with the COVID-19 preventive measures period, impacts air quality in Macao.

The levels of PM_2.5_ concentrations significantly reduced after the first confirmed case of COVID-19 pandemic in Macao on January 22nd, 2020, which caused panic and anxiety in the local population, and continued by the announcement of casino closures by the Macao government as part of the preventive measures for COVID-19 from February 5th to 20th, 2020. As some of the preventive measures, in particular, the 15 days mandatory casino closure have been lifted, the fear and tension of the local residents has eased, which has promoted population mobility. Although the levels of PM_2.5_ concentrations in Macao improved significantly during late January and early February 2020, the levels of PM_2.5_ concentrations gradually returned to normal in March 2020 after some of the preventive measures began to be lifted in Macao and its nearby Guangdong Province.

### 3.5. Air Quality Pollution Episodes Forecast

**Figure 7:**
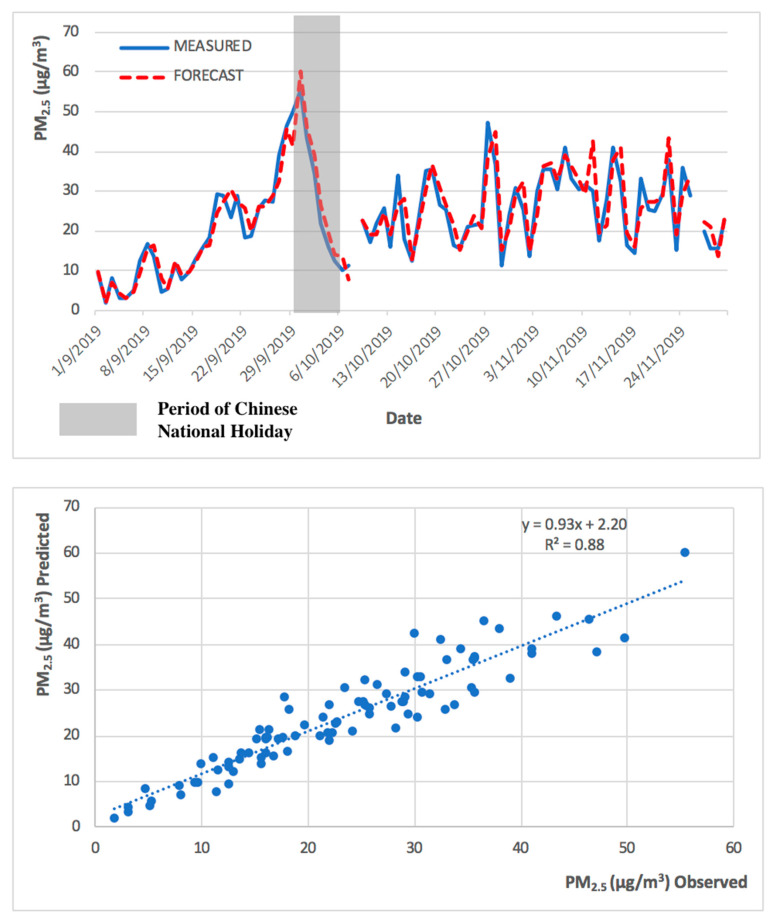
Observed and predicted PM_2.5_ concentrations for Taipa Ambient during Chinese National Holiday (from September to November 2019).

**Figure 8:**
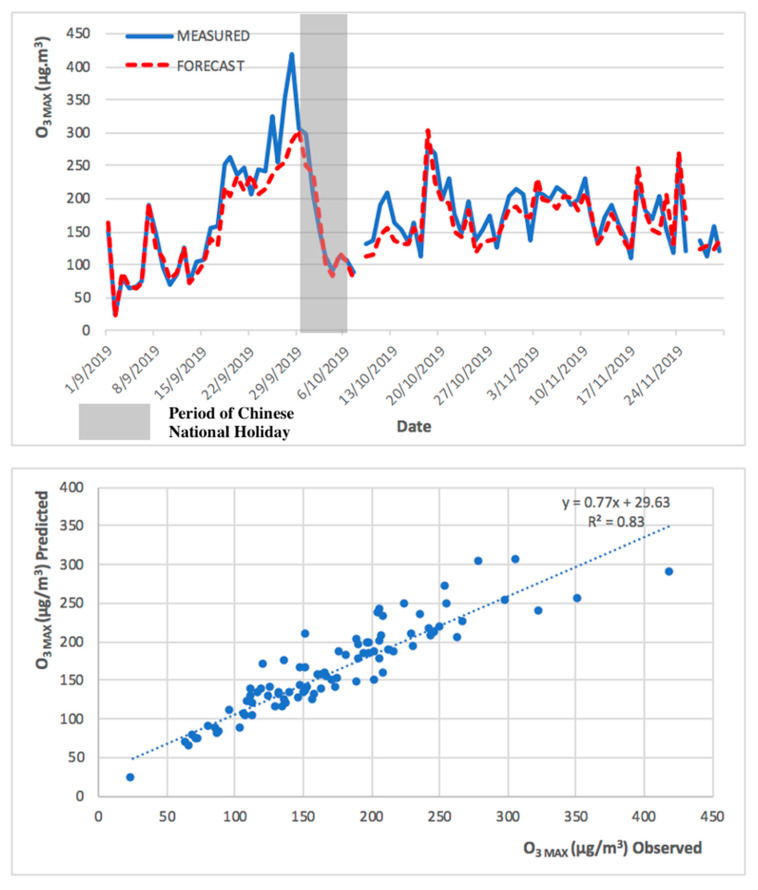
Observed and predicted O_3 MAX_ concentrations for Taipa Ambient during Chinese National Holiday (from September to November 2019).

Regarding the model behavior in predicting PM_2.5_ and O_3 MAX_ during the high pollution episode (Chinese National Holiday), observed and predicted PM_2.5_ and O_3 MAX_ concentrations are presented in Figure 7 and Figure 8.

As shown in Figure 7 and Figure 8, the levels of PM_2.5_ and O_3 MAX_ concentration peaked during late September and early October 2019. The PM_2.5_ predicted levels followed the primary trend of the measured concentrations and followed the concentration peak represented in Figure 7. The model for O_3 MAX_ also followed the primary trend, but it was more difficult to represent the concentration peak. The forecast model for PM_2.5_ has a higher R^2^ in comparison to the model of O_3 MAX_, because the maximum hourly concentration of O_3 MAX_ is more challenging to predict in comparison to the 24 h average of PM_2.5,_ as there is influence from the regional precursors sources and also its complex chemistry with solar radiation for O_3_ formation, which led to a higher degree of variability.

**Figure 9:**
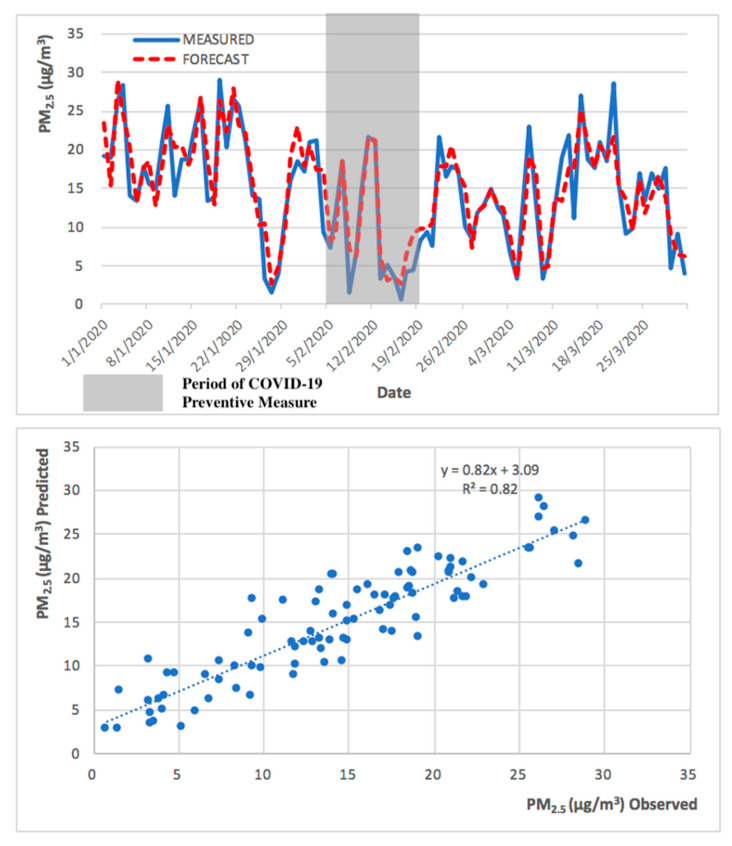
Observed and predicted PM_2.5_ concentrations for Taipa Ambient during preventive measures of COVID-19 pandemic (from January to March 2020).

**Figure 10:**
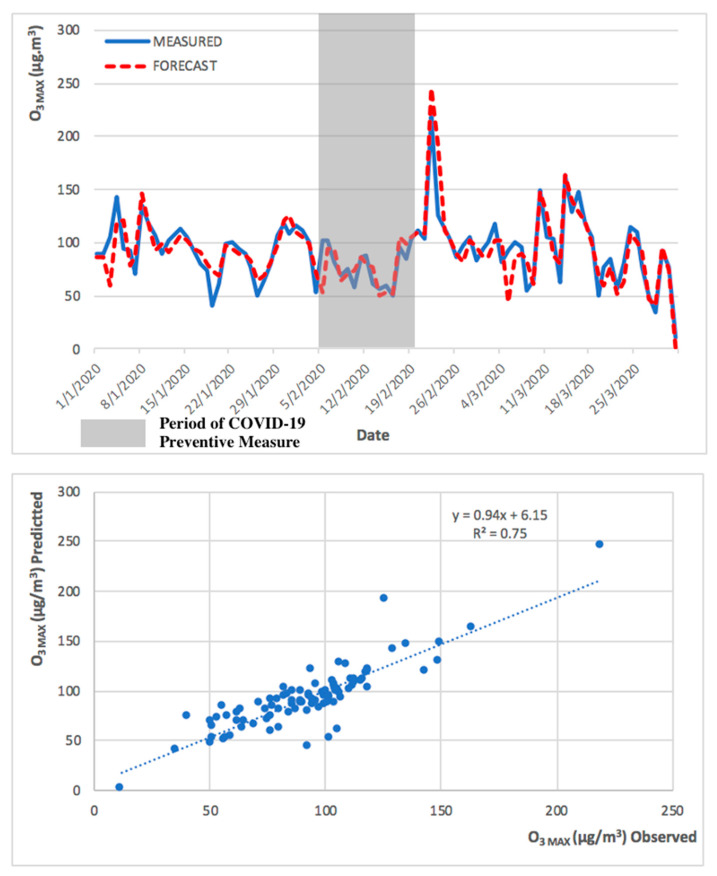
Observed and predicted O_3 MAX_ concentrations for Taipa Ambient during preventive measures of COVID-19 pandemic (from January to March 2020).

Due to the different nature of PM_2.5_ and O_3 MAX_, the forecast model performed better in the prediction of PM_2.5_ in comparison to O_3 MAX._ This can be demonstrated in the higher R^2^ values in the PM_2.5_ forecast model. The observed and predicted PM_2.5_ and O_3 MAX_ concentrations, during the low pollution episode (implementation of COVID-19 preventive measures), are presented in Figure 9 and Figure 10.

The 2013 to 2018 model successfully predicted both the high and low pollution episodes, for PM_2.5_ and O_3 MAX_, obtaining a significant R^2^ of 0.88 and 0.83, respectively, for the high pollution period (from September to November 2019), and an R^2^ of 0.82 and 0.75, respectively, for the low pollution period (from January to March 2020). The R^2^ obtained for the entire year of 2019 was 0.86 for both PM_2.5_ and O_3 MAX_. The statistical forecast model has been shown to be capable to predict, with a high coefficient of determination, the next 24 h.

## 4. Conclusions

As expected, the 2013 to 2018 model performed best with the highest R^2^ and lowest RMSE, MAE, and BIAS as compared with the 2013 to 2016 model and the 2015 to 2018 model. The additional two years of data helped to improve the accuracy and stability of the forecast of the 2013–2018 model.

The 2013–2018 model was able to successfully predict the high pollution episode during the Chinese National Holiday in late September and early October 2019 and the low pollution episode during the preventive measures period of COVID-19 pandemic in late January and early February 2020. This shows that this model can be reliably applied to forecast next-day pollutants concentrations across different magnitude levels of air pollution, being a useful tool for mitigation of air pollution impacts.

In addition, this shows that an improvement of global air quality in the territory is possible but it is tightly linked to the implementation of air pollution control measures in the industry and mobility sectors in Macao, in particular, in Guangdong Province. As previously studied, the air pollution problem associated with PM_2.5_ and O_3 MAX_ is a regional problem that is not only limited to Macao, but also in the nearby regions of Hong Kong and Guangdong Province.

## Figures and Tables

**Table 1:** Variables considered as predictors in the multiple linear regression (MLR) and classification and regression tree (CART) models in all of the air quality forecast models.

Variable Type	Variable Name	Variable Description (Units)/ Observations	
**Air quality variables**	NO_2_, PM_10_, PM_2.5_	Average hourly concentration values (µg/m^3^)	
	O_3 MAX_	Maximum hourly concentration values (µg/m^3^)	
	16D#, 23D#	23D#: 24-h concentration averaging period between 00h and 23h 16D#: 24-h concentration averaging period between 16h of D1 and 15h of D0 eg: PM10_16D1, O3_MAX_23D1.	
	D0, D1, D2, D3	D0: Forecast Day; D1: Previous Day (Forecast Day-1); D2: Forecast Day-2; and D3: Forecast Day-3.	
**Meteorological variables**	Upper-air obs. *	H1000, H850, H700, H500	Geopotential Height at 1000 hPa, 850 hPa, 700 hPa, and 500 hPa (m)/Indicator of synoptic-scale weather pattern.
		TAR925, TAR850, TAR700	Air Temperature at 925 hPa, 850 hPa, and 700 hPa (°C)/Measure of strength and height of the subsidence inversion.
		HR925, HR850, HR700	Relative Humidity at 925 hPa, 850 hPa, and 700 hPa (%).
		TD925, TD850, TD700	Dew Point Temperature at 925 hPa, 850 hPa, and 700 hPa (°C).
		THI850, THI700, THI500	Thickness at 850 hPa, 700 hPa, and 500 hPa (m)/Related to the mean temperature in the layer.
		STB925, STB850, STB700	Stability at 925 hPa, 850 hPa, and 700 hPa (°C)/Indicator of atmospheric stability.
**Surface observations**	T_AIR_MX, T_AIR_MD, T_AIR_MN	Maximum, Average, and Minimum Air Temperature (°C)	
	HRMX, HRMD, HRMN	Maximum, Average, and Minimum Relative Humidity (%)	
	TD_MD	Average dew point temperature (ground level) (°C)	
	RRTT	Precipitation (mm)/Associated with atmospheric washout	
	VMED	Average wind speed (m/s)/Related to dispersion	
**Other variables**	DD	Duration of the day: number of hours of sun per day (h)	
	FF	Week-day indicator (flag): weekday = 0, weekend = 1	

Meteorological variables: * Daily sounding at 12H (GMT+8) at King’s Park Meteorological Station—Hong Kong Observatory.

**Table 2:** Model performance indicators for the 2013 to 2016 model validation with 2019 data.

Station	Pollutant	Model Performance Indicator				Model Built Using Only MLR or CART and MLR	
		R^2^	RMSE	MAE	BIAS	MLR	CART
Macao Roadside	PM_10_	0.88	8.6	5.8	1.8	✓	
	PM_2.5_	0.86	5.4	3.7	1.5	✓	
	NO_2_	0.89	8.0	5.9	0.4	✓	
Macao Residential	PM_10_	0.89	8.8	5.9	−0.3	✓	
	PM_2.5_	0.87	5.2	3.3	0.7	✓	
	NO_2_	0.86	7.7	5.5	−0.4	✓	
	O_3 MAX_	0.85	23.2	14.0	0.0	✓	
Taipa Ambient	PM_10_	0.88	7.9	5.4	1.7	✓	
	PM_2.5_	0.86	5.1	3.6	1.6	✓	
	NO_2_	0.87	6.1	4.2	0.9	✓	
	O_3 MAX_	0.86	24.4	14.8	−2.1	✓	✓
Taipa Residential	PM_10_	0.87	8.0	5.2	0.1	✓	
	PM_2.5_	0.88	5.7	3.5	−0.1	✓	
	NO_2_	0.87	5.6	4.2	0.8	✓	
	O_3 MAX_	0.78	20.9	12.7	1.3	✓	✓
Coloane Ambient	PM_10_	0.88	8.7	6.2	2.4	✓	
	PM_25_	0.86	5.4	3.7	1.3	✓	
	NO_2_	0.81	7.8	5.5	−0.2	✓	
	O_3 MAX_	0.79	24.7	15.9	−3.6	✓	✓

**Table 3:** Model performance indicators for the 2013 to 2018 model validation with 2019 data.

Station	Pollutant	Model Performance Indicator				Model Built Using Only MLR or CART and MLR	
		R^2^	RMSE	MAE	BIAS	MLR	CART
Macao Roadside	PM_10_	0.88	8.4	5.6	1.5	✓	
	PM_2.5_	0.87	5.2	3.3	0.2	✓	
	NO_2_	0.89	7.9	5.8	−0.1	✓	
Macao Residential	PM_10_	0.89	8.8	5.9	−0.1	✓	
	PM_2.5_	0.87	5.2	3.3	0.8	✓	
	NO_2_	0.86	7.7	5.5	0.0	✓	
	O_3 MAX_	0.85	23.2	14.0	0.0	✓	
Taipa Ambient	PM_10_	0.88	7.8	5.1	0.8	✓	
	PM_2.5_	0.86	4.8	3.1	0.2	✓	
	NO_2_	0.87	6.1	4.2	1.0	✓	
	O_3 MAX_	0.86	23.7	14.7	−1.6	✓	✓
Taipa Residential	PM_10_	0.88	7.9	5.1	0.2	✓	
	PM_2.5_	0.88	5.6	3.5	−0.1	✓	
	NO_2_	0.87	5.6	4.1	0.6	✓	
	O_3 MAX_	0.78	20.9	12.7	1.3	✓	✓
Coloane Ambient	PM_10_	0.89	8.3	5.7	1.2	✓	
	PM_25_	0.86	5.3	3.6	1.0	✓	
	NO_2_	0.81	7.8	5.5	−0.1	✓	
	O_3 MAX_	0.79	24.3	15.3	–3.0	✓	✓

**Table 4:** Variables and model equations for each pollutant per air quality monitoring station in the 2013 to 2018 model.

Station	Pollutant	Model Equations
**Macao Roadside**	NO_2_	NO_2_ = 0.897 × NO_2__16D1 + 0.011 × H850 − 0.151 × HRMN
	PM_10_	PM_10_ = 0.913 × PM_10__16D1 + 0.015 × H850 − 0.208 × HRMD
	PM_2.5_	PM_2.5_ = 0.943 × PM_25__16D1 + 0.006 × H850 − 0.091 × HRMD
**Macao Residential**	NO_2_	NO_2_ = 0.913 × NO_2__16D1 + 0.007 × H850 − 0.087 × HRMN
	PM_10_	PM_10_ = 0.896 × PM_10__16D1 + 0.016 × H850 − 0.224 × HRMD
	PM_2.5_	PM_2.5_ = 0.926 × PM_25__16D1 + 0.004 × H850 − 0.176 × TD_MD
	O_3 MAX_	O_3 MAX_ = 1.089 × O_3_MAX__16D1 − 0.344 × O_3_MAX__23D1 − 1.303 × TD_MD + 1.437 × T_AIR_MX
**Taipa Ambient**	NO_2_	NO_2_ = 0.914 × NO_2__16D1 + 0.004 × H850 + 0.734 × STB925
	PM_10_	PM_10_ = 0.905 × PM_10__16D1 + 0.014 × H850 − 0.205 × HRMD
	PM_2.5_	PM_2.5_ = 0.928 × PM_25__16D1 + 0.006 × H850 − 0.093 × HRMD
	O_3 MAX_	If [O_3 MAX__16D1] ≤ 105.50 O_3 MAX_ = 1.034 × O_3_max__16D1 − 0.214 × O_3_max__23D1 + 0.019 × H850 − 0.236 × HRMN If [O_3 MAX__16D1] = ]105.50; 181.87] O_3 MAX_ = 0.994 × O_3_max__16D1 − 0.433 × O_3_max__23D1 + 0.051 × H850 − 0.529 × HRMN If [O_3 MAX__16D1] > 181.87 O_3 MAX_ = 1.006 × O_3_max__16D1 − 0.472 × O_3_max__23D1 + 0.12 × H850 − 2.025 × HRMN
**Taipa Residential**	NO_2_	NO_2_ = 0.859 × NO_2__16D1 + 0.007 × H850 − 0.271 × TD_MD
	PM_10_	PM_10_ = 0.902 × PM_10__16D1 + 0.015 × H850 − 0.204 × HRMD
	PM_2.5_	PM_2.5_ = 0.938 × PM_25__16D1 − 0.607 × TD_MD + 0.703 × TAR925
	O_3 MAX_	If [O_3 MAX__16D1] ≤ 129.12 O_3 MAX_ = 1.028 × O_3___max__16D1 − 0.238 × O_3_max__23D1 + 0.019 × H850 − 0.216 × HRMN If [O_3 MAX__16D1] = [129.12; 207.10] O_3 MAX_ = 0.958 × O_3_max__16D1 − 0.381 × O_3_max__23D1 + 0.061 × H850 − 0.751 × HRMN If [O_3 MAX__16D1] > 207.10 O_3 MAX_ = 1.12 × O_3_max__16D1 − 0.5 × O_3_max__23D1 + 0.14 × H850 − 2.818 × HRMN
**Coloane Ambient**	NO_2_	NO_2_ = 0.931 × NO_2__16D1 − 0.503 × TD_MD + 0.628 × TAR925
	PM_10_	PM_10_ = 0.904 × PM_10__16D1 + 0.015 × H850 − 0.214 × HRMD
	PM_2.5_	PM_2.5_ = 0.927 × PM_25__16D1 + 0.005 × H850 − 0.069 × HRMN
	O_3 MAX_	If [O_3 MAX__16D1] ≤ 116.20 O_3 MAX_ = 1.021 × O_3_max__16D1 − 0.233 × O_3_max__23D1 + 1.650 × T_AIR_MX − 1.392 × TD_MD If [O_3 MAX__16D1] = ]116.20; 186.92] O_3 MAX_ = 0.831 × O_3_max__16D1 − 0.397 × O_3_max__23D1 + 4.929 × T_AIR_MX − 3.384 × TD_MD If [O_3 MAX__16D1] > 186.92 O_3 MAX_ = 0.921 × O_3_max__16D1 − 0.482 × O_3_max__23D1 + 8.868 × T_AIR_MX − 8.582 × TD_MD
